# Replacement of Retinyl Esters by Polyunsaturated Triacylglycerol Species in Lipid Droplets of Hepatic Stellate Cells during Activation

**DOI:** 10.1371/journal.pone.0034945

**Published:** 2012-04-20

**Authors:** Nicole Testerink, Mokrish Ajat, Martin Houweling, Jos F. Brouwers, Vishnu V. Pully, Henk-Jan van Manen, Cees Otto, J. Bernd Helms, Arie B. Vaandrager

**Affiliations:** 1 Department of Biochemistry and Cell Biology, Faculty of Veterinary Sciences, Utrecht University, Utrecht, The Netherlands; 2 Medical Cell BioPhysics, MIRA Institute for Biomedical Technology and Technical Medicine, Department of Science and Technology, University of Twente, Enschede, The Netherlands; University of Bari & Consorzio Mario Negri Sud, Italy

## Abstract

Activation of hepatic stellate cells has been recognized as one of the first steps in liver injury and repair. During activation, hepatic stellate cells transform into myofibroblasts with concomitant loss of their lipid droplets (LDs) and production of excessive extracellular matrix. Here we aimed to obtain more insight in the dynamics and mechanism of LD loss. We have investigated the LD degradation processes in rat hepatic stellate cells *in vitro* with a combined approach of confocal Raman microspectroscopy and mass spectrometric analysis of lipids (lipidomics). Upon activation of the hepatic stellate cells, LDs reduce in size, but increase in number during the first 7 days, but the total volume of neutral lipids did not decrease. The LDs also migrate to cellular extensions in the first 7 days, before they disappear. In individual hepatic stellate cells. all LDs have a similar Raman spectrum, suggesting a similar lipid profile. However, Raman studies also showed that the retinyl esters are degraded more rapidly than the triacylglycerols upon activation. Lipidomic analyses confirmed that after 7 days in culture hepatic stellate cells have lost most of their retinyl esters, but not their triacylglycerols and cholesterol esters. Furthermore, we specifically observed a large increase in triacylglycerol-species containing polyunsaturated fatty acids, partly caused by an enhanced incorporation of exogenous arachidonic acid. These results reveal that lipid droplet degradation in activated hepatic stellate cells is a highly dynamic and regulated process. The rapid replacement of retinyl esters by polyunsaturated fatty acids in LDs suggests a role for both lipids or their derivatives like eicosanoids during hepatic stellate cell activation.

## Introduction

Hepatic stellate cells (HSCs) are non-parenchymal cells located perisinusoidally in the space of Disse and comprise about 5–10% of the total liver cell population [1]. HSCs play an important role in the turnover of hepatic extracellular matrix (ECM). They synthesize extracellular matrix proteins and secrete metalloproteinases to maintain the 3D structure of the liver in a dynamic way [2], [3]. During the process of liver injury and repair, HSCs become activated, and the quiescent HSC undergoes a gradual transformation from a non-dividing phenotype into a proliferative myofibroblastic phenotype [4], [5]. HSC activation and subsequent production of excessive ECM are therefore recognized as initial steps in the process of liver cirrhosis [6]. It is therefore important to understand the molecular mechanism that underlies the activation process of HSCs.

**Figure 1:**
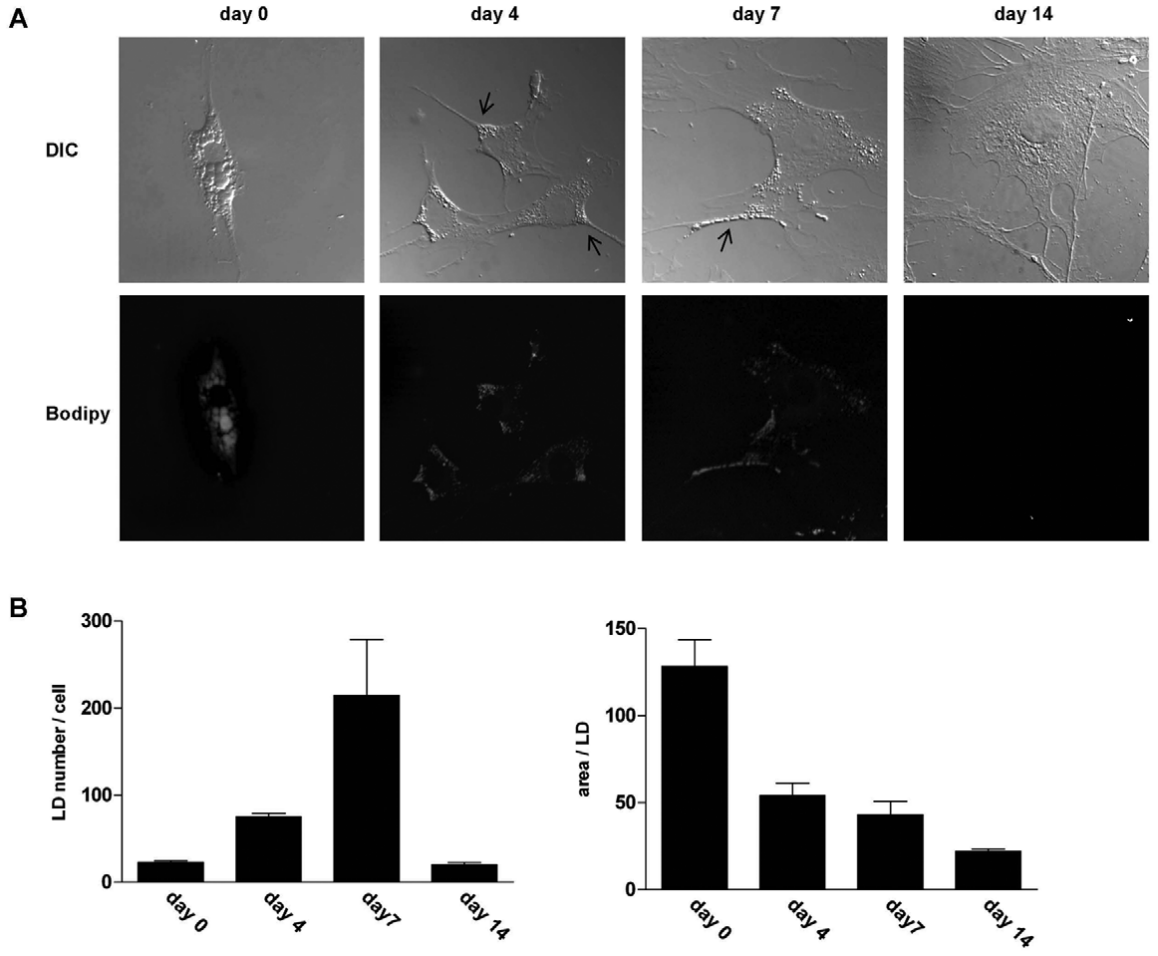
HSC activation results in redistribution of LDs and is accompanied by a decrease in size. **A.** Freshly isolated HSCs were cultured and fixed after 2 h (day 0), and 4, 7 or 14 days. Morphology and neutral lipid content was analyzed by differential interference contrast microscopy (DIC) and fluorescence microscopy after Bodipy staining of LDs. Arrows indicate LD redistribution. **B.** Total number of LDs in HSCs at day 0, 4, 7 and 14 and the area per LD was quantified by Image J software. The results represent the means ± SEM of 10 representative cells.

Quiescent HSCs have a lipid storing phenotype as indicated by the presence of large lipid droplets (LDs). During the activation process the HSCs lose their LDs [7]. LDs exist of a core of neutral lipids, surrounded by a phospholipid monolayer [8]. In most cells the neutral lipid stored in the LDs consists of triacylglycerols (TAG) and cholesterol esters. In HSCs, the LDs contain in addition to these neutral lipids also retinyl esters (RE). In fact, the surplus of retinol/vitamin A is mainly stored as RE in HSCs [9], [10]. The existence of two types of LDs is described in HSCs [10], although it is unknown whether one of these pools exclusively contains RE. Of the stored RE, retinyl palmitate is the most abundant species in rat HSC, followed by retinyl stearate and retinyl oleate [11]. The preferred esterification of retinol with saturated fatty acid species is mediated by the enzyme lecithin:retinol acyltransferase (LRAT) [12].

One of the unresolved issues in the field of HCS research is, whether the decrease in LDs is causally related to the activation process. In other words can HSC activation be altered when formation or breakdown of lipid droplets is disturbed? In order to answer this question first a more fundamental knowledge on the molecular mechanism of lipid droplet homeostasis in HSC is required as this is largely lacking at the moment. To acquire more insight in the mechanism of LD loss, and its role in HSC activation, we investigated the LD degradation process and lipidomic alterations in these cells with a combined approach of Raman confocal microspectroscopy and high performance liquid chromatography (HPLC)-coupled mass spectrometry (MS). Raman microspectroscopy - a spectroscopic technique based on inelastic scattering of monochromatic light - does not require labeling of the molecules of interest and enables direct specific chemical imaging of biomolecules such as DNA/RNA, proteins, and lipids in intact cells and tissues [13], [14]. More importantly, it provides detailed information about the molecular composition of the subcellular volume being probed [15]. Together with a newly developed MS technique enabling analysis of all neutral lipid classes in a single run, we could follow LDs morphologically and biochemically during the HSC activation process *in vitro*. Isolation of HSCs and subsequent culturing on a plastic surface induces phenotypical alterations resembling the *in vivo* HSC activation process and is therefore commonly used as a model to study HSC activation.

Here, we report that HSCs display a homogeneous LD population with respect to their chemical content. Upon activation LDs drastically change in size, intracellular location and lipidome. Especially a preferential decrease in retinyl esters was observed in the early stages of activation that was accompanied by a specific increase in triglycerides containing polyunsaturated fatty acids. This shows that lipid droplet degradation in activated hepatic stellate cells is a highly dynamic and regulated process.

## Results

### Redistribution of lipid droplets during HSC activation

To investigate the dynamics of the LD degradation process, freshly isolated HSCs were cultured and fixed at several time points, where after LD morphology and distribution was analysed. In its quiescent form, the HSC is relatively small and star-shaped (Fig. 1A-day 0). During the activation process, the HSC forms cellular extensions (Fig. 1A-day7 arrow), until its final transformation into a myofibroblastic cell (Fig. 1A-day 14). α-smooth muscle actin staining, commonly used as a HSC activation marker, confirmed the activated state of HSCs at day 7 and 14 (not shown). In the quiescent HSC numerous large LDs were observed perinuclearly (Fig. 1A-day 0). In HSCs around day 4 after plating an increased number of smaller LDs was visible, compared to quiescent cells. LDs were abundantly present in the many (newly formed) cell extensions. Quantification of LD size and number by Image J analysis confirmed that the size of the LDs decreased during the first days of activation, and remained similar between day 4 and 7 after plating, whereas the number of LDs continually increased (Fig. 1B). From these data, it can be calculated that the total volume of neutral lipids per cell did not decrease during the first 7 days. In the successive period the LDs were completely degraded, as 2 weeks after isolation hardly any LDs could be observed in the myofibroblastic HSCs (Fig. 1A-day14, 1B).

To investigate the fate of individual lipids in the LDs upon HSC activation in more detail, freshly isolated HSCs were incubated for 5 hr with 25 µM Bodipy-C12, a fluorescent fatty acid which is rapidly incorporated into TAG. After the incubation period, fluorescent fatty acid was accumulated in perinuclear LDs (Fig. S1). HSCs were subsequently cultured in the absence of exogenous Bodipy-C12 to follow the incorporated lipid in time. At day 4, fluorescently labelled LDs were detected at the cell extensions (Fig. S1). At day 7 fluorescent LDs were still observed at the growing cell tips (not shown), suggesting that the small LDs were at least partly derived from the larger perinuclear droplets rather than completely *de novo* formed from (unlabeled) lipids imported from the medium.

**Figure 2:**
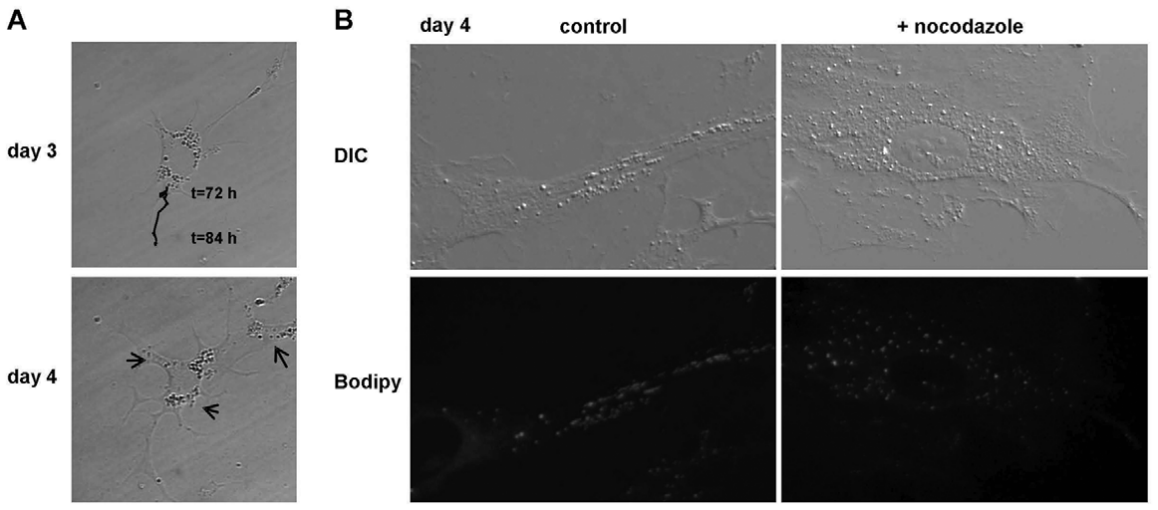
LD redistribution during HSC activation is microtubule dependent. **A.** Frames from time lapse life cell analysis revealing redistribution of dynamic LDs towards growing cellular extensions. Tracking shows the typical movements of a dynamic LD from 72 h to 84 h of HSC activation. Arrows indicate other regions of dynamic LDs (trackings not shown). **B.** To examine microtubule involvement in the LD redistribution process, freshly isolated HSCs were after 24 h in culture either treated with 10 µM nocodazole or vehicle (control) for 72 h at 37°C and after fixation, morphology and LD localization were analyzed by differential interference contrast microscopy (DIC) and fluorescence microscopy after Bodipy staining.

The notion that small peripheral LDs are derived from the large perinuclear LDs predicts that the LDs migrate to the cell extensions of activated HSCs. We therefore performed time-lapse live cell imaging on HSCs around day 3–4 after plating. At this time point the most dramatic alterations were observed in LD localization and cellular reshaping. As shown in Fig. 2A and Movie S1, migration of perinuclear LDs towards the growing cell extensions was apparent. LD redistribution could be disturbed by treatment with nocodazole, an agent affecting microtubule organisation, and LDs were scattered through the cytoplasm (Fig. 2B). This suggests that redistribution of the LDs towards the cell extensions is mediated by the microtubule system. In addition, nocodazole treated cells had less clear cell extensions in comparison with the control cells.

In order to obtain insight in the mechanism of the observed changes in LD number and size, we determined the level of a number of proteins implicated in LD formation in HSCs at day 1 and after activation at day 7 (See Fig. S2). We found minor changes in the level of diacylglycerol acyltransferase 2 (DGAT2), implicated in TAG synthesis. In contrast, LRAT, an enzyme responsible for the synthesis of RE, was down regulated during HSC activation. The level of a key enzyme in phosphatidylcholine (PC) synthesis, CTP:phosphocholine cytidylyltransferase alpha (CCTα) was somewhat lower in activated HSCs. The subcellular localization of CCTα as determined by immunofluorescence microscopy, was not different between day 1 and day 7 and was mostly nuclear (data not shown),

### Raman spectrum of retinyl esters is changed in activated HSC

**Figure 3:**
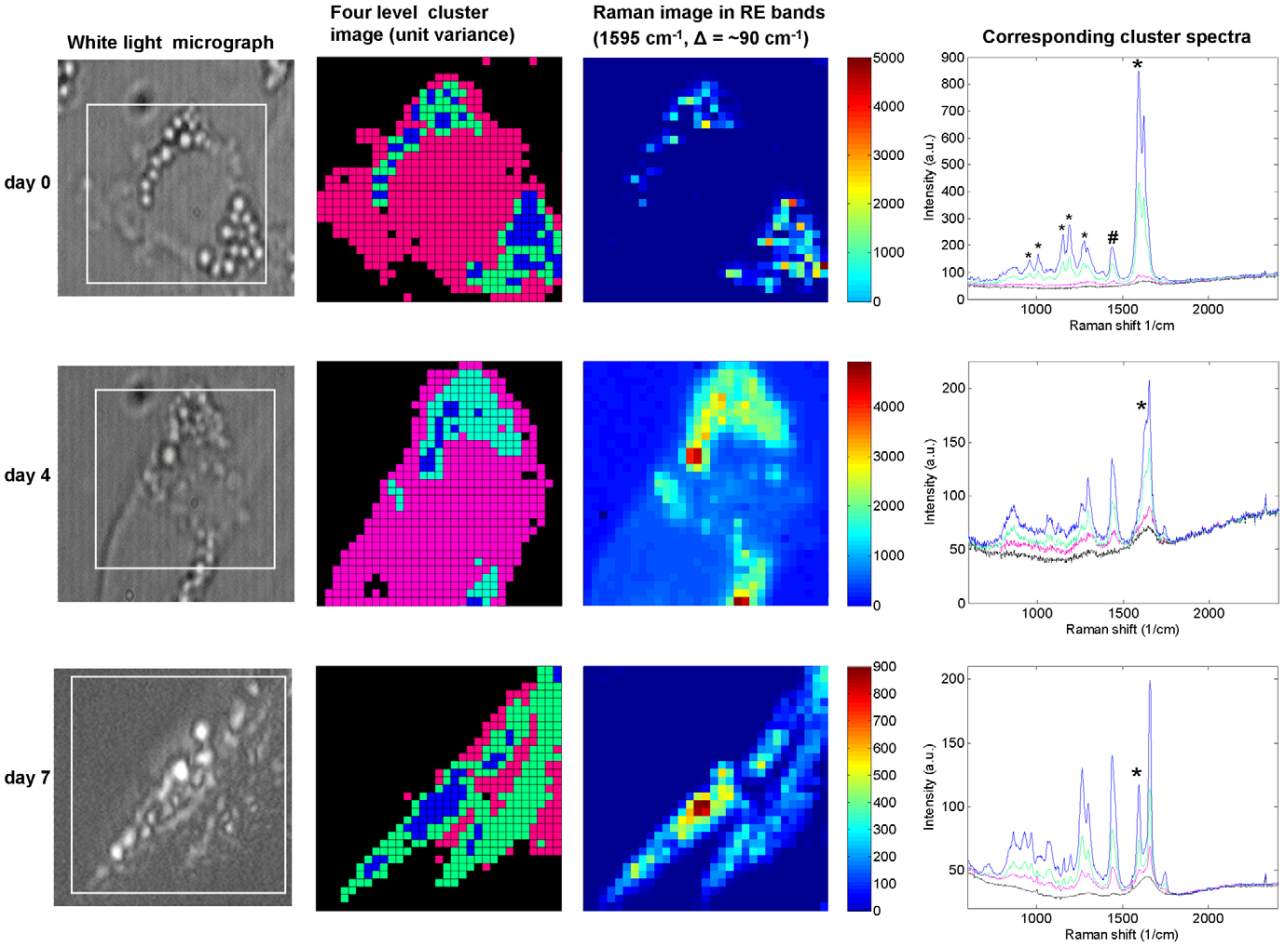
HSC activation results in a decrease in retinyl esters in HSC LDs. Freshly isolated HSCs were cultured and fixed after 2 h (day 0; quiescent state), and day 4 and day 7 (activated state). Confocal Raman microscpectroscopy on LD enriched regions was performed as described in the Method section. Cluster image (20×20 µm^2^) was constructed from Raman imaging data of the square area in the white light image. Each color represents a different cluster. The cluster averages show the average Raman spectra extracted from the black, pink, green and blue clusters displayed in the cluster image. * indicates (characteristic) RE peaks; # indicates characteristic acyl peak.

During the activation process the localisation and size of the LDs altered dramatically. To address whether the LD composition changed as well, confocal Raman microspectroscopy was used to investigate the LD composition of HSCs in the quiescent and activated state. Due to its conjugated structure, retinoids display a specific Raman spectrum. As seen in Fig. 3 (upper right panel) RE are abundantly present in quiescent HSCs, with a characteristic main peak at 1595 cm^−1^ and a number of smaller peaks in the region between 900 and 1300 cm^−1^ (marked with asterisks). Also the acyl chains of fatty acid containing lipids like TAG could be well detected by their characteristic peak at 1440 cm^−1^ (C-H bending; marked with #) and a peak around 1660 cm^−1^, which is partly obscured in the presence of the main retinyl peak, if present. By determining the ratio between the main retinyl peak (1595 cm^−1^) and the acyl peak at 1440 cm^−1^ both in HSC samples and in different mixtures of trioleylglycerol and retinyl palmitate standards, we could make an estimate of the retinyl content of the LDs. By this method, we found that approximately 10% of the LD content of quiescent HSCs consisted of retinylesters. However upon activation of the HSCs, a drastic decrease in the level of RE relative to triacylglycerol was seen already on day 4, which was even further decreased in the activated cells at day 7 (Fig. 3).

### (Activated) HSCs contain a homogenous LD population

In the initial period of the activation process both large perinuclear LDs and small dynamic LDs in cell extensions could be observed. To investigate whether the lipid composition of both LD types was similar, confocal Raman microspectroscopy was performed on perinuclear and peripheral LDs in HSCs at day 0, 4 and 7. As can be seen in the cluster images in Fig. 3 the Raman spectra of all LDs at day 0 contain a similar profile, with the same ratio between the peak at 1595 cm^−1^ (retinyl ester) and 1440 cm^−1^ (TAG species). Also at day 4 and day 7 all LDs within one cell showed a similar spectrum, although as described above, different from that in quiescent HSCs. (Fig. 3; lower panels). These results indicate that HSCs contain a homogenous LD population with respect to their neutral lipid composition, which is independent of their size, their state of activation and/or localization.

**Figure 4:**
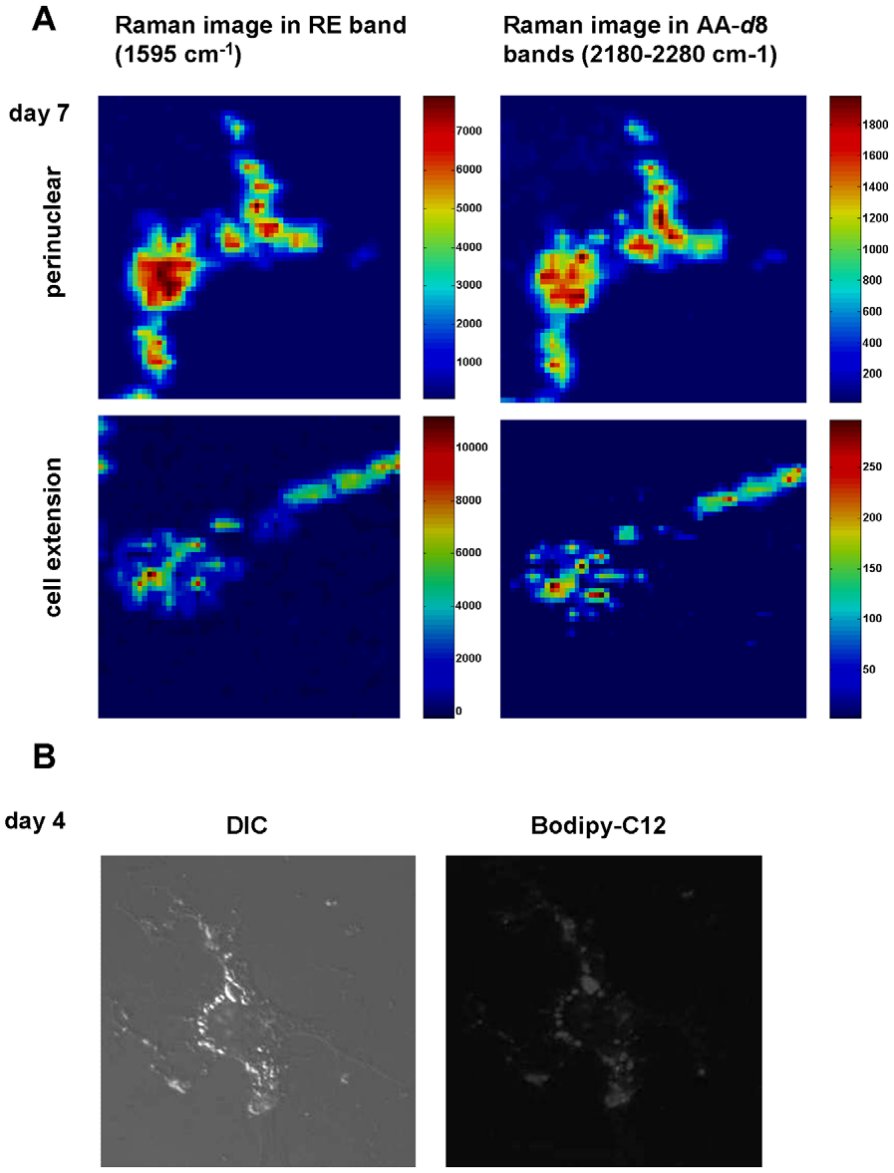
HSCs contain a metabolically homogenous population of LDs. **A.** Freshly isolated HSCs were cultured for 6 days and additionally incubated with 25 µM deuterated arachidonic acid (AA-*d*8) for another 24 h. After fixation confocal Raman microspectroscopy was performed as described. Raman images in the 1595 cm^−1^ (RE) and 2180–2280 cm^−1^ (AA-*d*8) regions are shown in arbitrary units for LD enriched sites perinuclearly (upper panels) and at the cell extension (from a different cell; lower panels). **B.** To determine metabolic activity of HSC LDs, freshly isolated HSCs were cultured for 4 days and subsequently incubated for 5 h with 25 µM Bodipy C-12. After fixation cells were analyzed by fluorescence microscopy.

To measure the metabolic activity of the LDs, we applied deuterated arachidonic acid (20:4-*d*8/AA-d8; 25 µM) to HSCs. Deuteration allowed the discrimination of exogenously administered fatty acids from endogenous lipids by a characteristic peak around 2200–2300 cm^−1^ of the C-D bond in the Raman spectrum [16]. All LDs at day 7 display a similar localization of the characteristic deuterium signal and the retinyl signal (Fig. 4A). This indicates that AA-*d*8 was incorporated in all LDs, both perinuclear and in the cell extensions. To obtain independent evidence for a similar metabolic activity of all LDs within one cell, we added the fluorescently labelled fatty acid, Bodipy-C12, to activated HSCs. Within one cell all LDs had incorporated similar levels of fluorescent fatty acid (Fig. 4B), although we could observe some differences in uptake between different cells plated at the same time.

### HSC activation results in a decrease in retinyl esters and an induction of polyunsaturated triacylglycerol species

**Figure 5:**
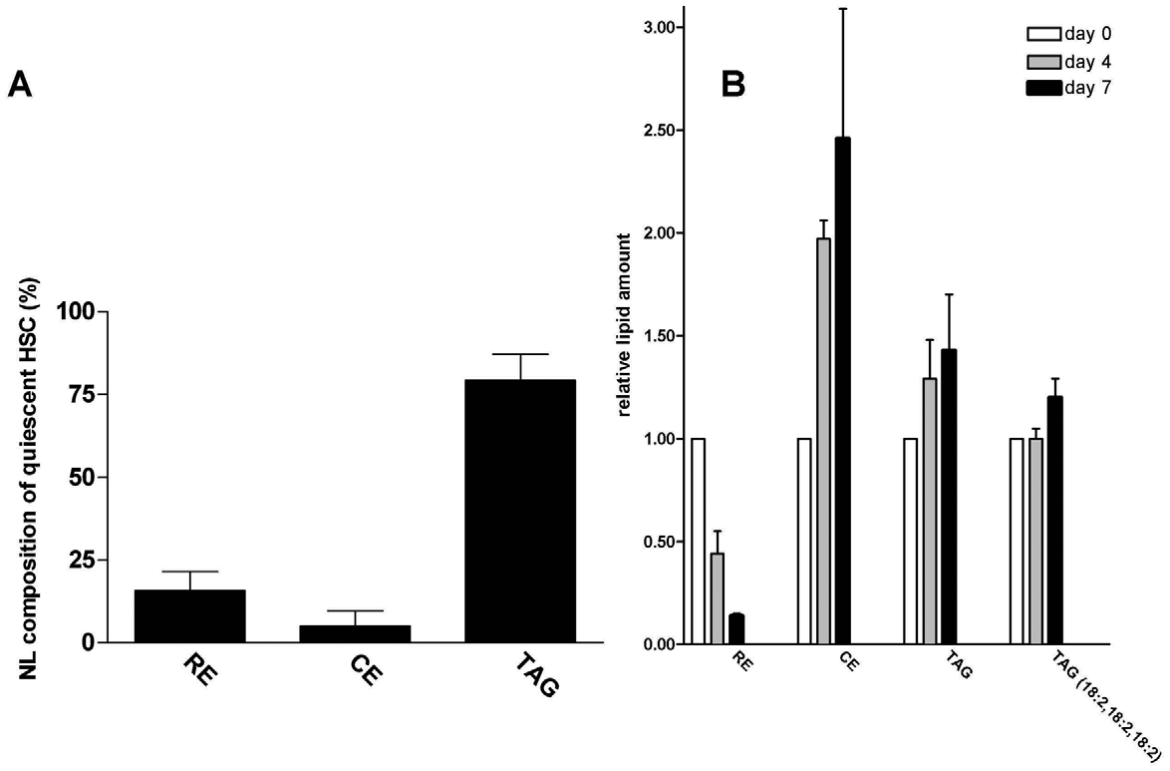
HSC activation results in a preferential decrease in retinyl esters. **A.** Neutral lipid composition of quiescent HSCs (day 0) analyzed by HPLC-APCI-MS. The results represent the means ± SEM of three experiments. **B.** Quantification of RE, cholesterol esters (CE), total TAG (TAG), and TAG(18:2,18-2,18:2) content in HSC at day 0, 4 and 7. Values are expressed relative to the level of lipid present at day 0. The results represent the means ± SEM of three experiments.

**Figure 6:**
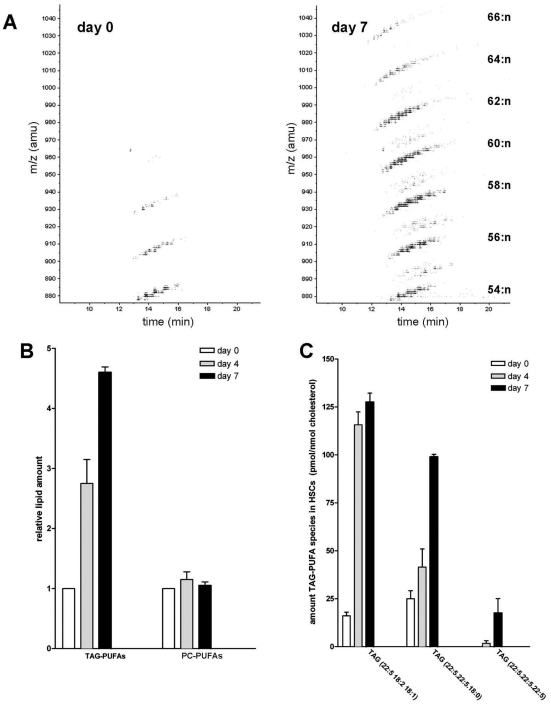
PUFA-containing TAG species, but not phospholipid species, are induced during HSC activation. **A.** Contour plots of HPLC-APCI-MS analysis of HSC at day 0 and 7, showing an increase in long chain fatty acid-containing TAG species at day 7. From every ion in the *m/z* 880–1050 region its relative abundance (amount of blackness) and retention time in the HPLC separation is shown. TAG species with the same total number of carbon atoms in the three acyl chains (denoted on the right hand side), but different number of double bonds (:n) form diagonal “stripes” at specific m/z regions. **B.** Quantification of the total amount of TAG-PUFA (*m/z* 900–1050) and PC-PUFA species in HSCs at day 0, 4 and 7. Values are expressed relative to the level of lipid present at day 0. The results represent the means ± SEM of three experiments. **C.** TAG species containing one, two or three 22:5 acyl moieties are induced during HSC activation. The results represent the means ± SEM of three experiments.

Our results suggest the presence of highly dynamic and metabolically active LDs. Therefore, we considered the possibility that both the lipid class profile and the lipid species (i.e. fatty acid) profile within a lipid class changed during HSC activation. We analysed the neutral lipid species profile of quiescent and activated HSC by HPLC-MS. We observed that 15–20% of the neutral lipids consisted of RE in quiescent HSCs (Fig. 5A). The main RE species was retinyl palmitate (68±8%), followed by retinyl stearate (17±4%). The remainder being retinyl linoleate and retinyl oleate (7±3% and 9±3%, respectively; n = 3). In accordance with the Raman experiments, a strong decline in the RE amount (mainly retinyl palmitate) was seen on day 4 after plating, and this decline continued upon further activation (Fig. 5B). However, activated cells had a somewhat increased level of TAG and a 2–3 fold higher level of cholesterol esters (Fig. 5B). Surprisingly, in activated HSCs TAG species with relatively high *m/z* values between 900–1050 were much more enriched, than species with lower *m/z* values like TAG (18:2,18:2,18:2), (Figs. 5B, 6A and 6B). Further analysis by MS/MS of the TAG species that were elevated in activated HSCs showed that most of the increased high *m/z* species had one or more long chain polyunsaturated fatty acids (PUFA) incorporated i.e. fatty acids of 20 and 22 carbon chain length and 4 or more double bonds. The most predominant PUFA was docosapentaenoic acid (22:5). Quantification of the TAG species containing two or three PUFAs (TAG-PUFAs) revealed that they were hardly detectable in quiescent HSC (Fig. 6C), but increased more than 10-fold upon activation. Especially TAG species with three PUFA moieties were specific for activated HSC at day 7 (Fig. 6C). After prolonged culture for one month, no TAG-PUFAs were present any more (data not shown).

HPLC-MS analysis of the phospholipid fractions of HSCs at day 0, 4 and 7 revealed no increase in PUFA species in phosphatidylcholine (PC), the most abundant phospholipid, upon activation (Fig. 6B). For a detailed PC-PUFA analysis see Fig. S3. Similar results were observed for phosphatidylethanolamine (PE), phosphatidylinositol (PI) and phosphatidylserine (PS) species (not shown). Hence, the increase in PUFA species is specific for TAG and not observed in phospholipids.

### Incorporation of exogenous arachidonic acid is increased during HSC activation

**Figure 7:**
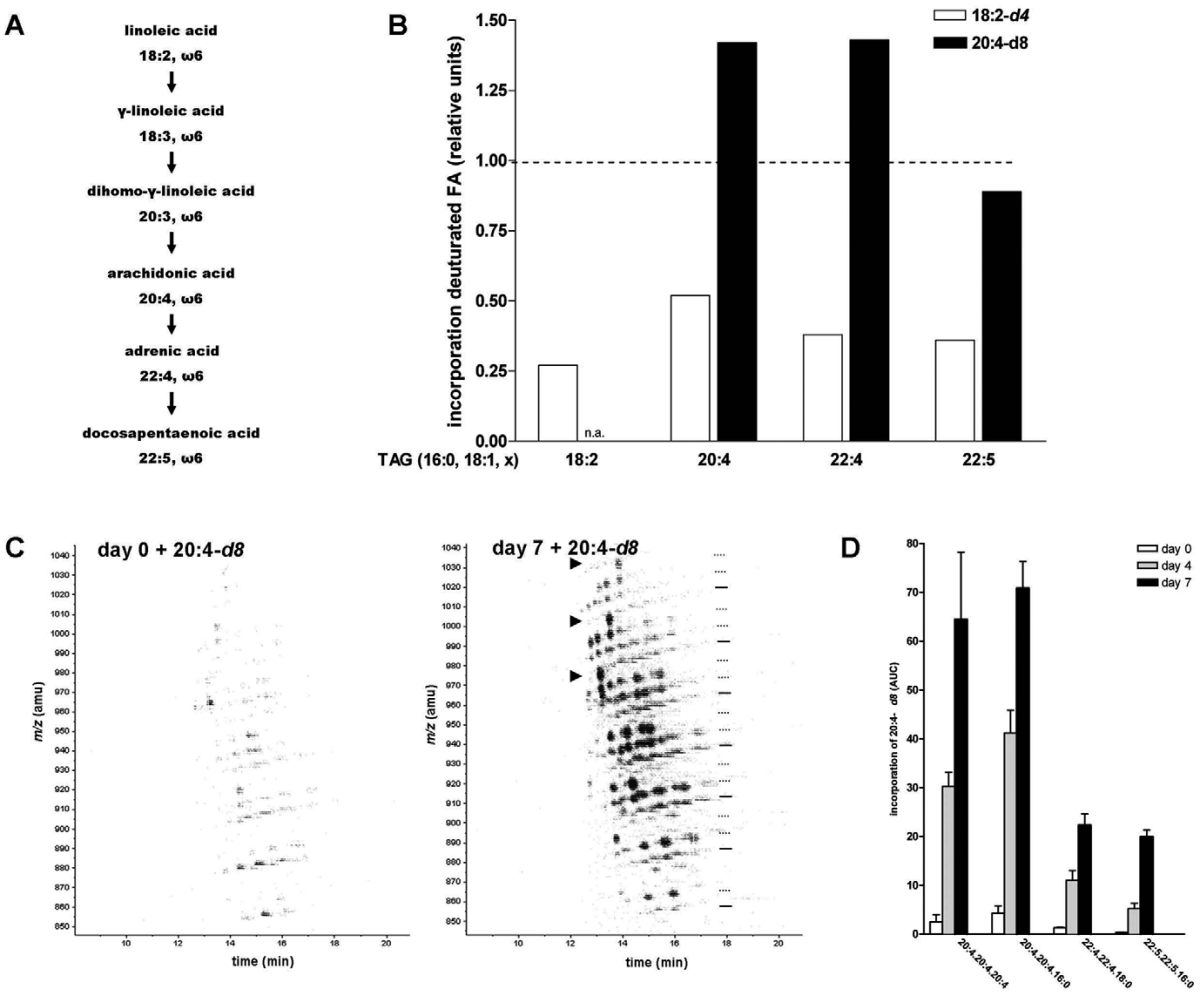
Incorporation of exogenous arachidonic acid into TAG is increased in activated HSCs. **A.** Overview of the omega-6 fatty acid synthesis pathway. **B.** Analysis of deuterated 18:2 and 20:4 incorporation in several TAG-PUFA species. Freshly isolated HSCs were incubated for seven days with 25 µM 18:2-*d4* or 25 µM 20:4-*d8*. After neutral lipid extraction, HPLC-MS was performed as described. Values are expressed relative to the level of the respective undeuturated TAG species present at day 7. The results represent the means ± SEM of three experiments. **C, D.** Freshly isolated HSCs were incubated at day 0, 3 or 6 with 25 µM 20:4-*d8* for 24 h. Subsequently, neutral lipids were extracted and HPLC-APCI-MS analysis was performed. **C.** Contour plots of HPLC-APCI-MS analysis of HSC at day 0 and 7 in the *m/z* 880–1050 region. Positions of unlabeled species are indicated with a straight line and relative position of species with one or two *d*8-labeled acyl chains are indicated by dotted lines. Arrowheads indicate TAG species with three *d*8-labeled acyl chains. **D.** Quantification of several TAG species containing *d8*-labeled acyl chains during HSC activation. The results represent the means ± SEM of three experiments.

PUFAs can be imported into cells or synthesized from various precursors by elongation and desaturation, starting from linoleic acid (18:2) or linolenic acid (18:3; for an overview of the PUFA (22:5) synthesis pathway see Fig. 7A). To investigate the origin of the observed TAG-PUFAs species, freshly isolated HSCs were incubated with deuterated linoleic acid (18:2-*d*4) or arachidonic acid (20:4-*d*8) for 7 days. Analysis of TAG species by HPLC-MS showed a higher incorporation of 20:4-*d*8 in comparison with 18:2-*d*4, (Fig. 7B). Not only 20:4-*d*8 containing TAG species were increased, but deuterated 22:4 and 22:5 species were also present, indicating elongation and desaturation of the imported fatty acids (Fig. 7B). As arachidonic acid was preferentially incorporated, we studied its incorporation and subsequent conversion in closer detail during the HSC activation process. HSCs in different states of activation were incubated for 24 h with 20:4-*d*8 (Fig. 7C). Whereas quiescent cells incorporate only minor quantities 20:4-*d8* in 24 h, the incorporation in activated cells was drastically increased (Fig. 7C and D). At day 7, activated HSCs incorporated 15–20 times more deuterated lipid than HSCs at day 1, in particular in the 20:4, 22:4 and 22:5 containing TAG species (Fig. 7D).

## Discussion

Raman microspectroscopy and HPLC-MS showed that the relative retinyl ester content in quiescent HSCs amounted to 10% and 15–20%, respectively, of the neutral lipid fraction. The somewhat lower percentage RE obtained by Raman measurements might be explained by laser-induced photo-oxidation effects of retinoids, as described by other groups [17]. Our findings indicate a lower percentage RE than the approximately 40–45% described in other studies, but a similar species profile (i.e. retinyl palmitate>>retinyl stearate>retinyl oleate≈retinyl linoleate) [11], [18]. It has been reported that the HSC LD composition is significantly influenced by the dietary retinol intake and age [19]. The rats in our experiments were fed a comparable amount of retinol (5 µg retinol/g dry food) and had similar weights (around 400 g) as described in the most frequently cited papers. However, the detection method for retinoids and other lipids differed between this study and the previous ones [11], [18].

Upon activation HSC were thought to start loosing both their RE and their LDs simultaneously. Based on our observations we suggest that LD turnover during HSC activation occurs in two phases. Initially, LDs redistribute from perinuclear regions towards newly formed cellular extensions. During this first phase the total LD volume does not decrease, but the LDs reduce in size and increase in number. This might indicate fission of LDs during the redistribution process. Fission of LDs has been observed recently in fission yeast during cell cycle [20], although the mechanism of LD fission remained obscure. Interestingly, rat HSCs are reported to upregulate cell cycle proteins and DNA synthesis upon activation [21]. The increase in the number of LDs also suggests an increase in the amount of phospholipids, mainly PC, surrounding the LDs. CTP:phosphocholine cytidylyltransferase (CCT), a key enzyme involved in PC synthesis, was recently shown to be required for the increase in LD number upon oleate addition to mammalian cells [22]. However, we could not observe an increase or translocation of CCTα in activated rat HSCs, suggesting that the existing CCTα levels suffice for the generation of enough PC for the phospholipid monolayers covering the novel LDs and for the newly formed cell extensions. The translocation of the LDs towards the cell extensions probably involves directed movement along microtubules as it was inhibited by nocadozole. The signals and mechanism of LD movements are largely unknown, but might involve specific regulation of a core motor machinery [23].

In addition to changes in LD localization and morphology, the LD lipid content alters as well. The RE concentration declines considerably and TAG species containing PUFAs rise to relatively high levels. The loss of RE, approximately 15% of total lipids in LDs, is roughly similar to the amount of newly formed PUFA-TAGs. So RE are apparently replaced by PUFA-TAGs in LDs during the first phase. In the second phase of the LD degradation process, the small LDs reduce further in size until hardly any LDs are present in the activated, myofibroblastic HSC.

As demonstrated by Raman microspectroscopy no differences in lipid composition between individual LDs within one cell were observed, both in quiescent and activated HSCs. This indicates that the intracellular LD population behaves homogeneously with respect to RE degradation and other lipid metabolic pathways. The molecular identity of the enzymes involved in LD degradation in HSCs during activation is largely unexplored. Adipose triglyceride lipase (ATGL) has an important role in LD degradation in eukaryotic cells [24]. A contribution of hormone sensitive lipase (HSL), an enzyme involved in LD degradation in adipocytes, is less likely as this enzyme is relatively adipocyte specific and has not been detected in hepatic stellate cells. Also several proteins are implicated in RE hydrolysis, including hepatocyte carboxylesterase ES-3, ES-4, ES-10 and ES-22 [25], [26]. Recently, autophagy was found to play a role in the degradation of lipids in activated mouse and human HSC's [27], implicating lysosomal lipases in the degradation process of LDs. More extended proteomic screening during HSC activation may reveal enzymes associated with the degradation/conversion of cholesterol esters, TAGs and RE and unravel their roles in HSC activation.

We demonstrated that the levels of TAGs and RE were not decreased simultaneously upon HSC activation, suggesting a specific lipolytic regulation for these two classes of lipids. Alternatively, the lipid content (both TAGs and RE) of existing LDs may be degraded simultaneously, but the resulting retinol and fatty acids may be re-esterified at different rates. In support of this latter possibility it was shown that in activated HSCs the enzyme involved in esterification of retinol, i.e. LRAT, was more down regulated than DGAT2, an enzyme involved in incorporation of fatty acids into TAG [28] (Fig. S2).

The observation that RE are degraded in the initial phase of HSC activation suggests a role for the released retinol or its metabolites like retinoic acid, during liver injury and/or repair. Recently, HSCs devoid of retinoid containing LDs, were shown to have a similar effect on liver fibrosis as normal RE containing HSCs, but caused a decrease in hepatic carcinogenesis [29].

Together with the loss of RE in the initial phase of HSC activation, we demonstrated an enhanced incorporation of exogenous arachidonic acid (AA) in TAG and formation of TAG-PUFA species. The selective incorporation of PUFAs in TAG implies the involvement of either i) specific transporters for PUFAs, ii) enzymes which selectively couple PUFAs to CoAs or iii) enzymes which selectively incorporate PUFA-CoAs into TAG. Among the various enzymes known to be involved in these pathways only the long-chain acyl-CoA synthetase 4 (ACSL4) was described to be selective for AA and other C20 fatty acids [30]. However, no large increase in mRNA levels of this enzyme was observed during HSC activation (unpublished observation; [31]).

Elevation of TAG-PUFA species during HSC activation could be physiologically relevant in at least two different cellular processes. First, long chain PUFAs are known to be abundantly present in membrane phospholipids of sperm and neuronal cells, affecting membrane fluidity and regulating the function of several membrane associated proteins, including those involved in fusion of transport organelles with the plasma membrane [32]–[33]. Neurons and spermatocytes have in common that they have long cellular extensions, similar to activated HSC. As during transdifferentiation from quiescent HSC to myofibroblast long extensions are formed, phospholipids have to be synthesized to increase both the membrane surface area and the fluidity of the extensions. Although phospholipid analysis of total cell homogenates did not show an enhancement of PUFA containing phospholipid species in activated HSCs, specific enrichment of PL-PUFAs in the plasma membranes of activated HSCs cannot be excluded.

A second process in which the increase in TAG-PUFAs may play a role is the AA/eicosanoid metabolism. AA plays a central role in inflammation and is involved in many pathological conditions [34]. Cubero and colleagues report that in alcoholic liver disease AA can accelerate ECM synthesis by HSCs, resulting in liver cirrhosis [35]. AA is also a precursor for eicosanoids, which are signaling lipids that play a role in a broad range of processes, such as modulation of the immune system. In several types of immune cells esterified AA has been found in isolated LDs [36]. It has been suggested that in leukocytes such LDs serve as an AA reservoir for local activation of essential cellular functions [16]. Also proteins involved in AA metabolism and transport were found on the surface of LDs supporting the hypothesis that LDs might have a central role in eicosanoid synthesis and secretion [36]. The HSC has also been recognized as antigen presenting cell involved in a range of immunological functions [37]. TAG-PUFAs found in our lipidomic screen could therefore be storage pools, waiting to be incorporated in phospholipids or used for local eicosanoid synthesis, as described in immune cells.

In conclusion, we demonstrated that drastic lipidomic changes take place during early HSC activation, including preferential RE depletion and an increase in the formation of TAG-PUFA species. These lipid species proved to be very characteristic markers for HSCs in their initial activation state and underline the importance of the lipidome during (patho)physiological alterations.

## Materials and Methods

### Ethics statement

Rats were treated according to the strict governmental and international guidelines on animal experimentation, and were approved by the Animal Experimentation Committee (Dierexperimentencommissie; DEC) of Utrecht University (DEC-numbers: 2009.III.08.076 and 2010.III.09.110).

### Chemicals

Collagenase (Clostridium histolyticum Type I) was obtained from Sigma-Aldrich (St. Louis, MO, USA). Dulbecco's modified Eagles's medium (DMEM) and fetal bovine serum (FBS) were from Gibco (Grand Island, NY, USA). Bodipy 558/568-C12 (D-3835) and Bodipy 493/503 (D-3922) were from Molecular Probes (Invitrogen, Carlsbad, CA, USA). Antibody against glial fibrillary acidic protein (GFAP) was from BD Pharmingen (San Jose, CA, USA), against α-smooth muscle actin (α-SMA) from Thermo Scientific (Waltham, MA, USA), against diacylglycerol acyltransferase 2 (DGAT2) and β-actin from Abcam (Cambridge, MA, USA), against lecithin:retinol acyltransferase (LRAT) from Santa Cruz Biotechnology (Santa Cruz, CA, USA), and against CTP:phosphocholine cytidylyltransferase alpha (CCTα) from Cell Signaling Technology (Danvers, MA, USA), All HPLC-MS solvents were from Biosolve (Valkenswaard, the Netherlands) with exception of chloroform (Carl Roth, Karlsruhe, Germany) and were of HPLC grade. Silica-G (0,063–0,200 mm) was purchased from Merck (Darmstadt, Germany). Diacylglycerol (16:0,16:0), TAG (18:1, 18:1, 18:1), cholesterol, CE (18:1), and retinyl palmitate standards were from Sigma-Aldrich (St. Louis, USA). Linoleic acid-*d4* (18:2-*d*4) and arachidonic acid-*d8* (20:4-*d*8/AA- *d*8) were purchased from Cayman Chemical (Ann Arbor, MI, USA).

### Isolation and culture of HSC

Adult male Wistar rats (350–400 g) were used in all experiments. Stellate cells were isolated from rat liver by collagenase digestion followed by differential centrifugation [38]. Cell isolations were performed under reduced light conditions to prevent retinoid oxidation. After isolation, HSC were plated in 25 cm^2^ culture flasks or on glass coverslips. Purity of the plated cell population was checked routinely by immuno-staining of glial fibrillary acidic protein (GFAP). Cells were maintained in Dulbecco's modified Eagles's medium (DMEM) supplemented with 10% fetal bovine serum (FBS), 100-units/ml penicillin and 100 µg/ml streptomycin and 4 µl/ml Fungizone. Medium was changed every 4 days. Cells were protected from light exposure by wrapping culture flasks and dishes in aluminium foil.

### Fluorescence and live cell microscopy

Freshly isolated HSCs were grown on glass coverslips. Cells were fixed at day 0, 4, 7 and 14 with 4% paraformaldehyde in phosphate-buffered saline (PBS). For staining of the LDs, cells were incubated with Bodipy 493/503 (final concentration 0.02 µg/ml) for 15 min at room temperature. Specimens were analyzed using a LEICA DMR fluorescence microscope. Images were analysed using Image J software. For the analysis lipid loss from LDs during the activation process, freshly isolated HSCs were incubated with 25 µM Bodipy 558/568-C12 for 5 h at 37°C. After washing to remove the excess of fluorescent lipid, cell culture was continued, and cells were fixed with 4% paraformaldehyde at several time points. To study the dynamics of lipid incorporation into LDs during the HSC activation process, cells at day 0, 4 and 7 were incubated with 25 µM Bodipy 558/568-C12 for 5 h at 37°C and fixed with 4% paraformaldehyde. Specimens were analyzed using a LEICA DMR fluorescence microscope.

For live cell imaging, cells were cultured on Fluorodisk wells and placed in a Solent scientific cell incubator with temperature and CO_2_ control. Life cell analysis was performed for 12 hours from 72 h to 84 h after plating Time lapse series were obtained using a BioRad Radiance 2100 MP confocal system (Zeiss/BioRad, Hertfordshire, UK).

### Confocal Raman microspectroscopy and imaging

Freshly isolated HSCs were grown on calcium fluoride cover slips and fixed at day 0, 4 and 7 with 2% paraformaldehyde in PBS for 15 minutes. In some experiments cells at day 0, 3 and 6 were incubated with 25 µM deuturated arachidonic acid (20:4-*d8*) for 24 h, whereafter they were fixed. For calibration of the RE and TAG detection pure lipid standards (retinyl palmitate and trioleylglycerol) and mixtures of several ratios hereof were used. Non-resonant Raman spectroscopy and imaging experiments were performed on a laser-scanning confocal Raman microspectrometer [13]. Imaging experiments were performed by raster-scanning the laser beam over a LD or an intracellular region of interest and accumulating a full Raman spectrum at each pixel (1 s/pixel at 23 mW 647.1-nm excitation power). Noise in the resulting 3D (spatial×spatial×spectral dimension) data matrix was reduced by singular value decomposition [13], [16]. Raman images were constructed by plotting the integrated intensity of the vibrational band of interest as a function of position. Hierarchical cluster analysis (HCA) was performed on Raman imaging data matrices to visualize regions in cells with high Raman spectral similarities. In the cluster analysis routine, principal component analysis scores were taken as input variables, squared Euclidean distances were used as distance measure, and Ward's algorithm was used to partition Raman spectra into clusters. All data manipulations were performed in routines written in Matlab 6.5 (MathWorks, Natick, MA).

### High-performance liquid chromatography and mass spectrometry

Samples were kept under red light and all procedures were carried out in brown vials to prevent retinoid isomerization and oxidation. Lipids were extracted from equal protein amounts of total cell homogenate of HSC at day 0, 4, 7 and 1 month after isolation by the method of Bligh and Dyer [39]. Extracted lipids were separated in a neutral and phospholipid fraction by fractionation on a freshly prepared silica-G column (approximately 10 mg of 0,063–0,200 mm silica) [40]. Lipid extracts were dissolved in methanol/chloroform (1/9, v/v) and loaded on top of the silica column. Neutral lipids were eluted with two volumes acetone, dried under nitrogen gas and stored at −20°C. Just before HPLC-MS analysis, the neutral lipid fraction was reconstituted in methanol/chloroform (1/1, v/v) and separated on a Lichrospher RP18-e column (5 µm, 250×4.6 mm; Merck, Darmstadt, Germany). A gradient was generated from acetonitrile to acetone/chloroform 85/15, v/v, at a constant flow rate of 1 ml/min. Mass spectrometry of lipids was performed using Atmospheric Pressure Chemical Ionization (APCI) on a Biosystems API-4000 Q-trap (MDS Sciex, Concord, Canada). The system was controlled by Analyst version 1.4.2 software (MDS Sciex, Concord, ON, Canada) and operated in positive ion mode using the following settings: source temperature 420°C, nebulizer gas (GS1) 5, nebulizer current 3 µA, curtain gas 10, collision gas High and declustering potential 100 V. The optimal collision energy was dependent on the type of experiment and was set to +10 V (full scan mode) or +30 V (product ion mode). In all full scan runs spectra were obtained from *m/z* 250–1100.

To identify the exact composition of high m/z value TAG species, an enhanced product ion spectrum (optimal CE +35 V) was made of all ions with an m/z value over 850. The solvents and gradient used were similar as described above.

The phospholipid fraction was dissolved in methanol/acetonitrile/chloroform/water (46:20:17:17) and directly injected into the mass spectrometer. Mass spectrometry of phospholipids was performed using Electrospray Ionization (ESI) on a Biosystems API-4000 Q-trap. The system was controlled by Analyst version 1.4.2 software and operated in positive mode (phosphatidylcholine (PC), precursor scan of 184 *m/z* and phosphatidylethanolamine (PE), neutral loss of 141 amu) and negative mode (phosphatidylinositol (PI), precursor scan of 241 *m/z* and phosphatidylserine (PS), neutral loss of 87 amu), using the following settings: source temperature 450°C, nebulizer gas (GS1) 45, curtain gas 10 and collision gas High. Declustering potential and collision energy were depending on the type of experiment and were respectively for PC 120 V and 47 V, for PE 110 V and 30 V, for PI −110 V and −60 V and for PS −100 V and −36 V.

Data analysis was performed using Analyst 1.4.2 software (MDS Sciex, Concord, ON, Canada) and calibration curves of all lipid classes were established under similar conditions as the samples.

## Supporting Information

Figure S1 **Migration of perinuclear LDs towards the growing cell extensions in activated HSCs.** To investigate LD redistribution during HSC activation, freshly isolated HSCs were incubated at day 0 with 25 µM Bodipy C-12 for 5 h. After washing the excessive Bodipy C-12, cells were fixed (day 0) or cultured for 4 days in the absence of dye before fixation (day 4). Cells were analyzed by fluorescence microscopy.
(TIF)

Figure S2 **Changes in protein levels of various enzymes implicated in LD metabolism during HSC activation.** Western blots of equal amounts of total protein from isolated rat HSC one day after plating (dy1) and 7 days after plating (dy7). Blots were probed with antibodies against CTP:phosphocholine cytidylyltransferase alpha (CCTα), diacylglycerol acyltransferase 2 (DGAT2), lecithin:retinol acyltransferase (LRAT), and β-actin (ACT; loading control).
(TIF)

Figure S3 **Incorporation of PUFAs in phosphatidylcholine species is not increased during HSC activation.** Phospholipid extracts of HSCs harvested at day 0, 4 and 7 were analyzed by HPLC-MS as described. The results represent the means ± SEM of three experiments.
(TIF)

Movie S1 **LD dynamics in activated HSC.** Time lapse life cell analysis was performed for 12 h on a typical HSC from 72 h to 84 h after plating as described in method section. Frames were taken every 30 minutes.
(AVI)

## References

[1] Smedsrod B, De Bleser PJ, Braet F, Lovisetti P, Vanderkerken K (1994). Cell biology of liver endothelial and kupffer cells.. Gut.

[2] Clement B, Emonard H, Rissel M, Druguet M, Grimaud JA (1984). Cellular origin of collagen and fibronectin in the liver.. Cell Mol Biol.

[3] Friedman SL (2008). Hepatic stellate cells: Protean, multifunctional, and enigmatic cells of the liver.. Physiol Rev.

[4] Mathew J, Geerts A, Burt AD (1996). Pathobiology of hepatic stellate cells.. Hepatogastroenterology.

[5] Gressner AM (1998). The cell biology of liver fibrogenesis – an imbalance of proliferation, growth arrest and apoptosis of myofibroblasts.. Cell Tissue Res.

[6] Safadi R, Friedman SL (2002). Hepatic fibrosis – role of hepatic stellate cell activation.. MedGenMed.

[7] Friedman SL, Wei S, Blaner WS (1993). Retinol release by activated rat hepatic lipocytes: Regulation by kupffer cell-conditioned medium and PDGF.. Am J Physiol.

[8] Martin S, Parton RG (2006). Lipid droplets: A unified view of a dynamic organelle.. Nat Rev Mol Cell Biol.

[9] Hendriks HF, Verhoofstad WA, Brouwer A, de Leeuw AM, Knook DL (1985). Perisinusoidal fat-storing cells are the main vitamin A storage sites in rat liver.. Exp Cell Res.

[10] Blaner WS, O'Byrne SM, Wongsiriroj N, Kluwe J, D'Ambrosio DM (2009). Hepatic stellate cell lipid droplets: A specialized lipid droplet for retinoid storage.. Biochim Biophys Acta.

[11] Yamada M, Blaner WS, Soprano DR, Dixon JL, Kjeldbye HM (1987). Biochemical characteristics of isolated rat liver stellate cells.. Hepatology.

[12] Yost RW, Harrison EH, Ross AC (1988). Esterification by rat liver microsomes of retinol bound to cellular retinol-binding protein.. J Biol Chem.

[13] Puppels GJ, de Mul FF, Otto C, Greve J, Robert-Nicoud M (1990). Studying single living cells and chromosomes by confocal raman microspectroscopy.. Nature.

[14] Uzunbajakava N, Lenferink A, Kraan Y, Volokhina E, Vrensen G (2003). Nonresonant confocal raman imaging of DNA and protein distribution in apoptotic cells.. Biophys J.

[15] Hanlon EB, Manoharan R, Koo TW, Shafer KE, Motz JT (2000). Prospects for in vivo raman spectroscopy.. Phys Med Biol.

[16] van Manen HJ, Kraan YM, Roos D, Otto C (2005). Single-cell raman and fluorescence microscopy reveal the association of lipid bodies with phagosomes in leukocytes.. Proc Natl Acad Sci U S A.

[17] Failloux N, Bonnet I, Baron MH, Perrier E (2003). Quantitative analysis of vitamin A degradation by raman spectroscopy.. Appl Spectrosc.

[18] Hendriks HF, Blaner WS, Wennekers HM, Piantedosi R, Brouwer A (1988). Distributions of retinoids, retinoid-binding proteins and related parameters in different types of liver cells isolated from young and old rats.. Eur J Biochem.

[19] Moriwaki H, Blaner WS, Piantedosi R, Goodman DS (1988). Effects of dietary retinoid and triglyceride on the lipid composition of rat liver stellate cells and stellate cell lipid droplets.. J Lipid Res.

[20] Long AP, Manneschmidt AK, Verbrugge B, Dortch MR, Minkin SC (2012). Lipid droplet de novo formation and fission are linked to the cell cycle in fission yeast..

[21] Dudas J, Saile B, El-Armouche H, Aprigliano I, Ramadori G (2003). Endoreplication and polyploidy in primary culture of rat hepatic stellate cells.. Cell Tissue Res.

[22] Krahmer N, Guo Y, Wilfling F, Hilger M, Lingrell S (2011). Phosphatidylcholine synthesis for lipid droplet expansion is mediated by localized activation of CTP:Phosphocholine cytidylyltransferase.. Cell Metab.

[23] Welte MA (2009). Fat on the move: Intracellular motion of lipid droplets.. Biochem Soc Trans.

[24] Smirnova E, Goldberg EB, Makarova KS, Lin L, Brown WJ (2006). ATGL has a key role in lipid droplet/adiposome degradation in mammalian cells.. EMBO Rep.

[25] Mello T, Nakatsuka A, Fears S, Davis W, Tsukamoto H (2008). Expression of carboxylesterase and lipase genes in rat liver cell-types.. Biochem Biophys Res Commun.

[26] Schreiber R, Taschler U, Wolinski H, Seper A, Tamegger SN (2009). Esterase 22 and beta-glucuronidase hydrolyze retinoids in mouse liver.. J Lipid Res.

[27] Hernandez-Gea V, Ghiassi-Nejad Z, Rozenfeld R, Gordon R, Fiel MI (2012). Autophagy releases lipid that promotes fibrogenesis by activated hepatic stellate cells in mice and in human tissues..

[28] Yamaguchi K, Yang L, McCall S, Huang J, Yu XX (2008). Diacylglycerol acyltranferase 1 anti-sense oligonucleotides reduce hepatic fibrosis in mice with nonalcoholic steatohepatitis.. Hepatology.

[29] Kluwe J, Wongsiriroj N, Troeger JS, Gwak GY, Dapito DH (2011). Absence of hepatic stellate cell retinoid lipid droplets does not enhance hepatic fibrosis but decreases hepatic carcinogenesis.. Gut.

[30] Cao Y, Traer E, Zimmerman GA, McIntyre TM, Prescott SM (1998). Cloning, expression, and chromosomal localization of human long-chain fatty acid-CoA ligase 4 (FACL4).. Genomics.

[31] Xia Y, Chen R, Song Z, Ye S, Sun R (2010). Gene expression profiles during activation of cultured rat hepatic stellate cells by tumoral hepatocytes and fetal bovine serum.. J Cancer Res Clin Oncol.

[32] Rapoport SI (2001). In vivo fatty acid incorporation into brain phosholipids in relation to plasma availability, signal transduction and membrane remodeling.. J Mol Neurosci 16: 243–61; discussion.

[33] Darios F, Davletov B (2006). Omega-3 and omega-6 fatty acids stimulate cell membrane expansion by acting on syntaxin 3.. Nature.

[34] Harizi H, Corcuff JB, Gualde N (2008). Arachidonic-acid-derived eicosanoids: Roles in biology and immunopathology.. Trends Mol Med.

[35] Cubero FJ, Nieto N (2008). Ethanol and arachidonic acid synergize to activate kupffer cells and modulate the fibrogenic response via tumor necrosis factor alpha, reduced glutathione, and transforming growth factor beta-dependent mechanisms.. Hepatology.

[36] Bozza PT, Magalhaes KG, Weller PF (2009). Leukocyte lipid bodies – biogenesis and functions in inflammation.. Biochim Biophys Acta.

[37] Vinas O, Bataller R, Sancho-Bru P, Gines P, Berenguer C (2003). Human hepatic stellate cells show features of antigen-presenting cells and stimulate lymphocyte proliferation.. Hepatology.

[38] Riccalton-Banks L, Bhandari R, Fry J, Shakesheff KM (2003). A simple method for the simultaneous isolation of stellate cells and hepatocytes from rat liver tissue.. Mol Cell Biochem.

[39] Bligh EG, Dyer WJ (1959). A rapid method of total lipid extraction and purification.. Can J Biochem Physiol.

[40] Retra K, Bleijerveld OB, van Gestel RA, Tielens AG, van Hellemond JJ (2008). A simple and universal method for the separation and identification of phospholipid molecular species.. Rapid Commun Mass Spectrom.

