# Supplementary figures and images for: Replacement of Retinyl Esters by Polyunsaturated Triacylglycerol Species in Lipid Droplets of Hepatic Stellate Cells during Activation
Source: PLoS One. 2012 Apr 20;7(4):e34945. doi: 10.1371/journal.pone.0034945 (PMC3335019; doi:10.1371/journal.pone.0034945)

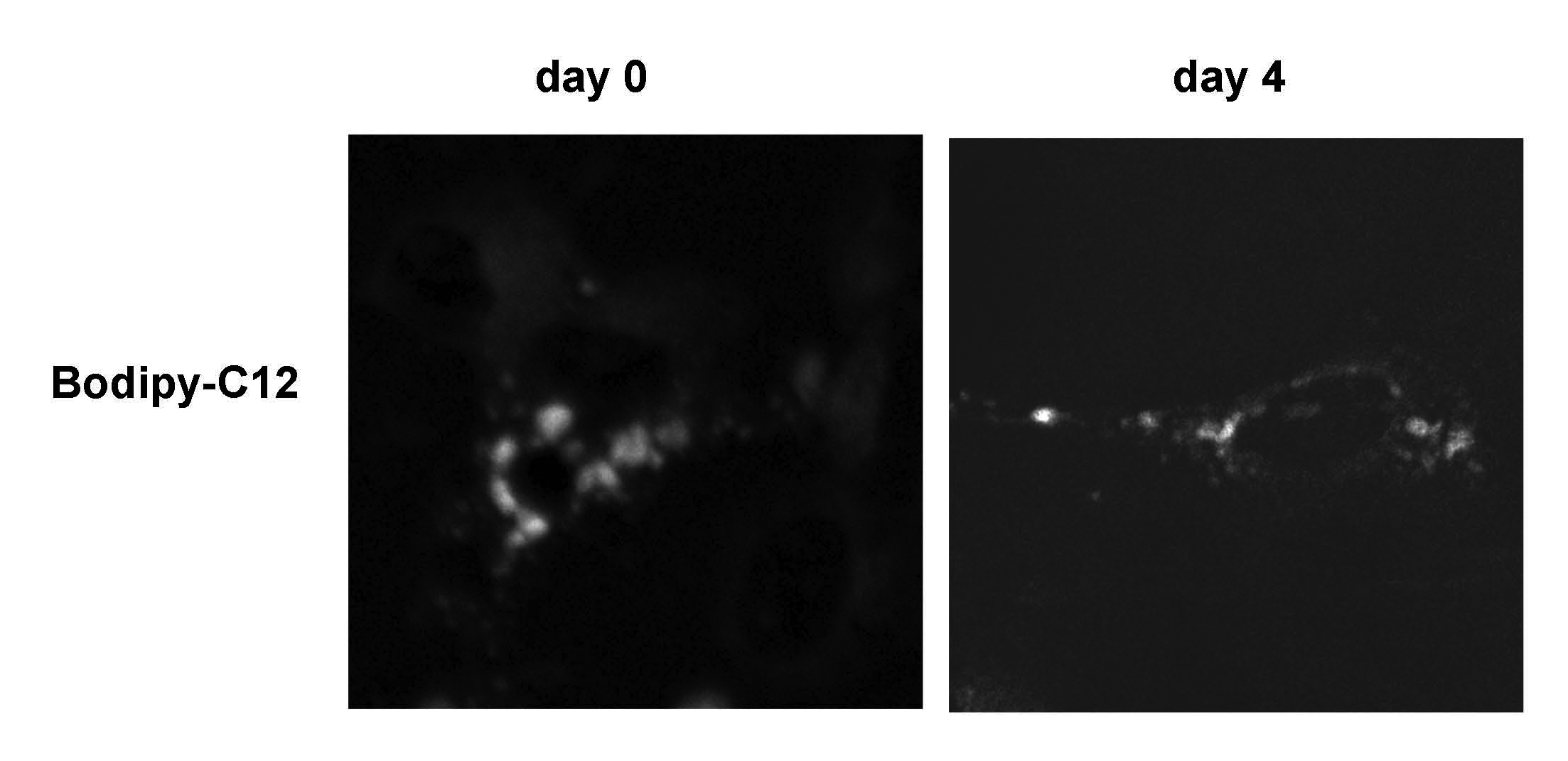

Supplement: Figure S1 — Migration of perinuclear LDs towards the growing cell extensions in activated HSCs. To investigate LD redistribution during HSC activation, freshly isolated HSCs were incubated at day 0 with 25 µM Bodipy C-12 for 5 h. After washing the excessive Bodipy C-12, cells were fixed (day 0) or cultured for 4 days in the absence of dye before fixation (day 4). Cells were analyzed by fluorescence microscopy. (TIF) [file pone.0034945.s001.tif]

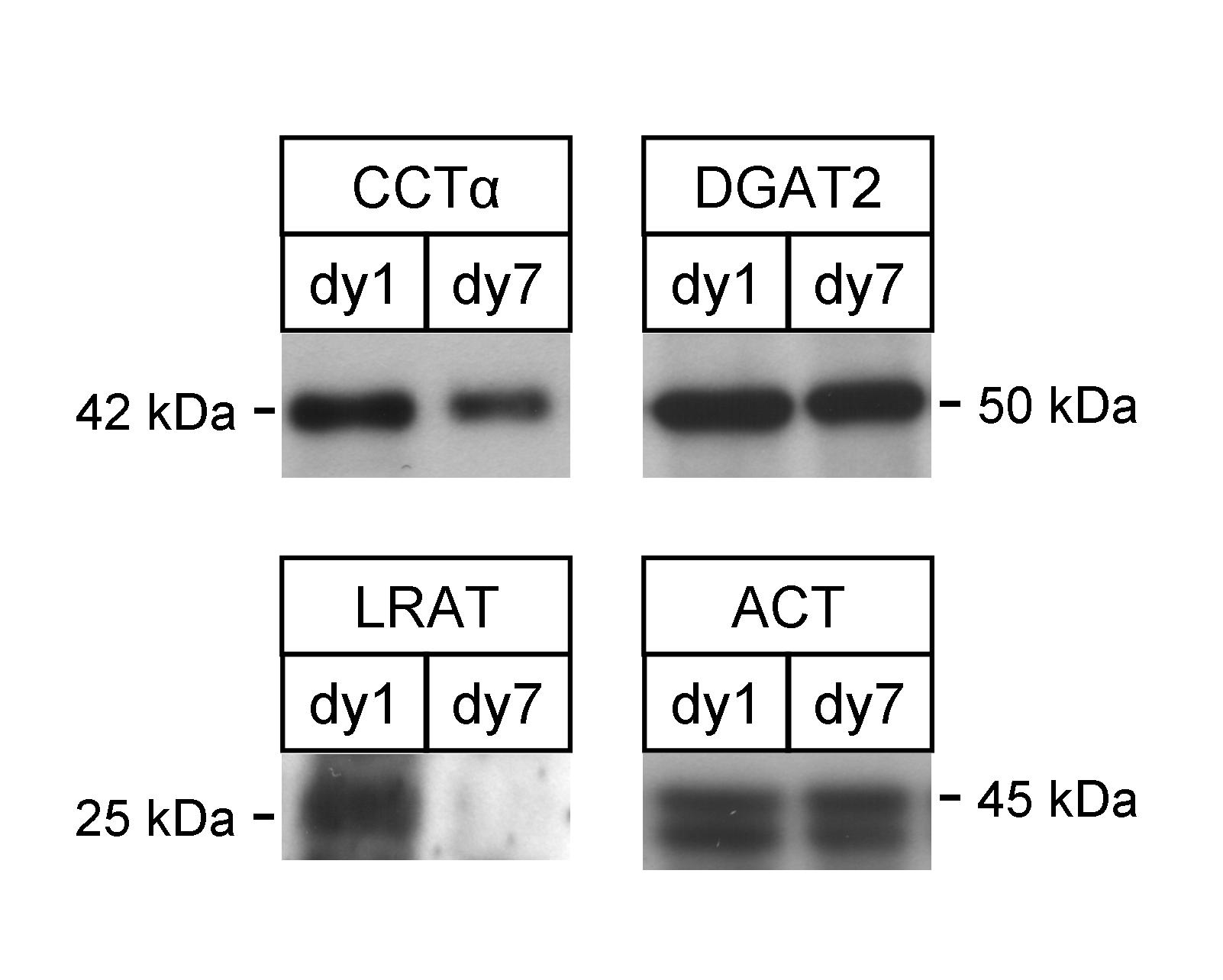

Supplement: Figure S2 — Changes in protein levels of various enzymes implicated in LD metabolism during HSC activation. Western blots of equal amounts of total protein from isolated rat HSC one day after plating (dy1) and 7 days after plating (dy7). Blots were probed with antibodies against CTP:phosphocholine cytidylyltransferase alpha (CCTα), diacylglycerol acyltransferase 2 (DGAT2), lecithin:retinol acyltransferase (LRAT), and β-actin (ACT; loading control). (TIF) [file pone.0034945.s002.tif]

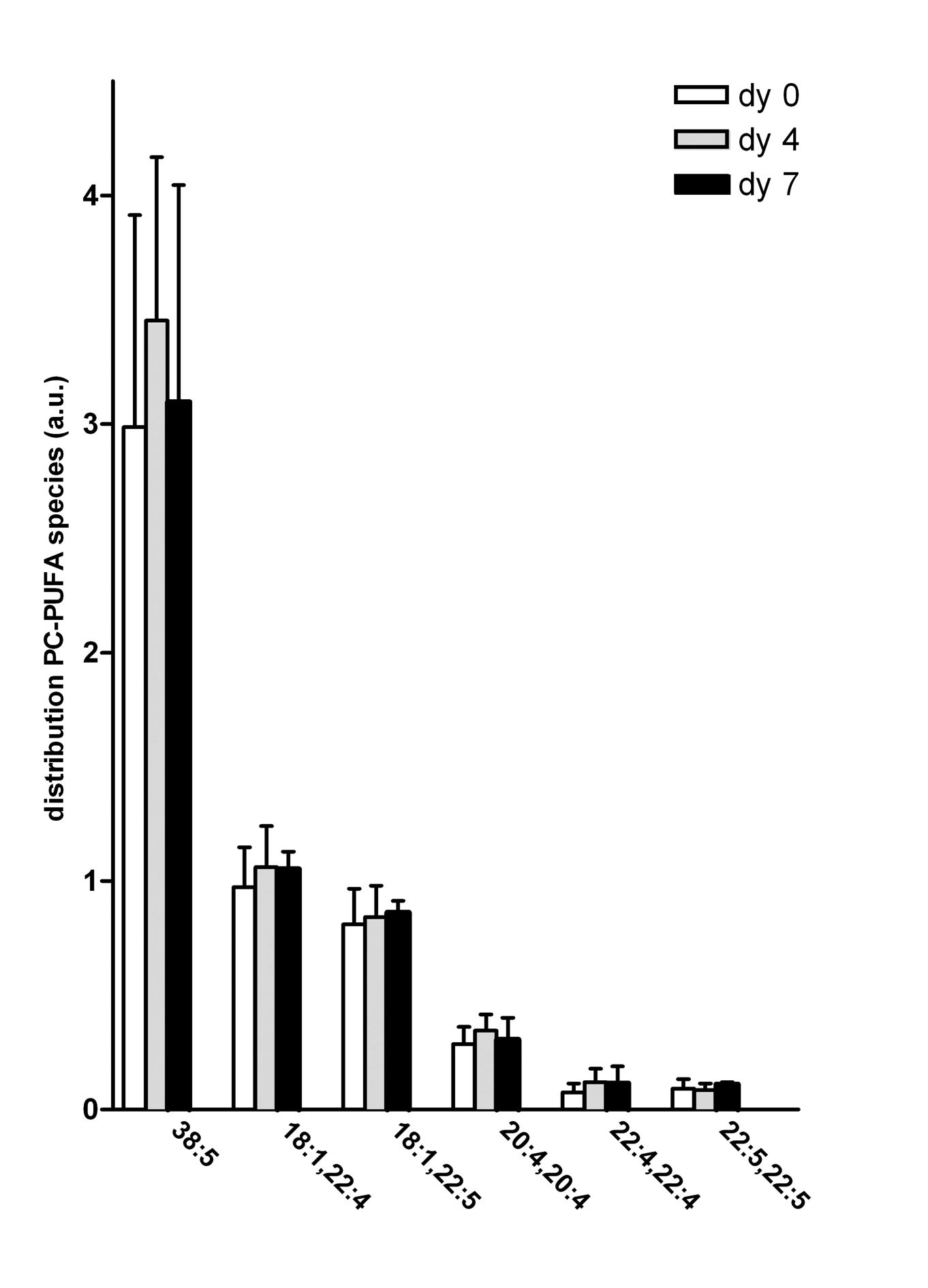

Supplement: Figure S3 — Incorporation of PUFAs in phosphatidylcholine species is not increased during HSC activation. Phospholipid extracts of HSCs harvested at day 0, 4 and 7 were analyzed by HPLC-MS as described. The results represent the means ± SEM of three experiments. (TIF) [file pone.0034945.s003.tif]
